# Supplementary material for: A deep-learning approach for online cell identification and trace extraction in functional two-photon calcium imaging
Source: Nat Commun. 2022 Mar 22;13:1529. doi: 10.1038/s41467-022-29180-0 (PMC8940911; doi:10.1038/s41467-022-29180-0)
Supplement: Supplementary file 1 — Supplementary Information [file 41467_2022_29180_MOESM1_ESM.pdf]

Supplementary Information

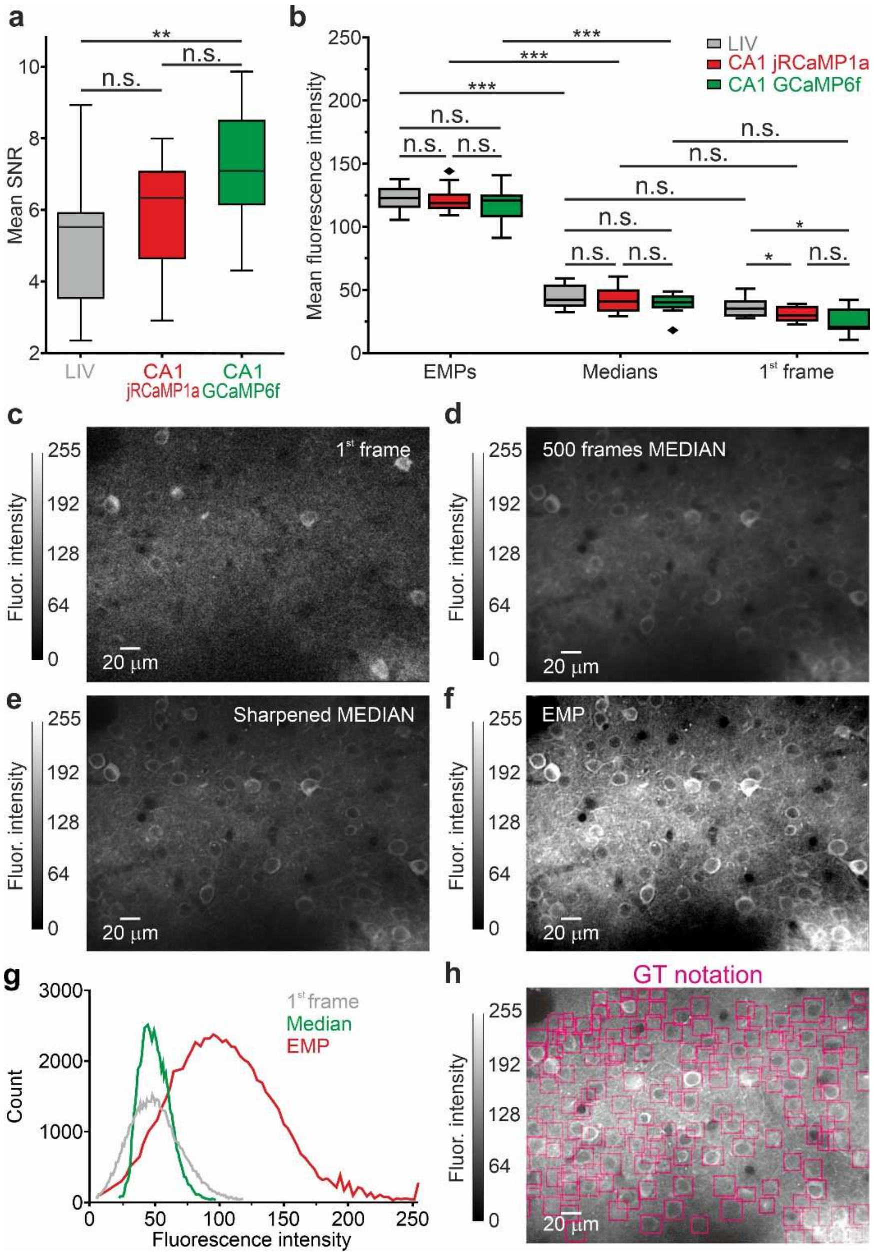

Supplementary Figure 1. Processing imaging t-series before Ground Truth (GT) annotation. a) SNR values across all the t-series in the dataset (both training and validation). Two-sided unpaired Student's *t*-test, $p = 0.17$ between LIV vs. CA1 jRCaMP1a; $p = 0.009$ for LIV vs. CA1 GCaMP6f; $p = 0.26$ for CA1 GCaMP6f vs. CA1 jRCaMP1a. $N = 121$ for LIV, $N = 33$ for CA1 jRCaMP1a and for CA1 GCaMP6f. **b)** Fluorescence intensity for EMPs images, median projections of time series and individual first frames of acquisition for LIV (grey) and CA1 (jRCaMP1a: red and GCaMP6f: green) datasets. For EMPs, unpaired Student's *t*-test: $p = 0.77$ for LIV vs. CA1 jRCaMP1a; $p = 0.62$ for LIV vs. CA1 GCaMP6f; $p = 0.78$ for CA1 jRCaMP1a vs. CA1 GCaMP6f. For median values, Wilcoxon rank sum test: $p = 0.28$ for LIV vs. CA1 jRCaMP1a; $p = 0.35$ for LIV vs. CA1 GCaMP6f; $p = 0.58$ for CA1 jRCaMP1a vs. CA1 GCaMP6f. For first individual frames, Wilcoxon rank sum test: $p = 0.038$ for LIV vs. CA1 jRCaMP1a; $p = 0.025$ for LIV vs. CA1 GCaMP6f; $p = 0.68$ for CA1 jRCaMP1a vs. CA1 GCaMP6f. For EMPs vs. median values, Wilcoxon sum rank test $p = 3.5E\text{-}5$ for

LIV EMP vs. LIV median; p = 5.6E-6 for CA1 jRCaMP1a EMP vs. CA1 jRCaMP1a median; p = 5.6E-6 for CA1 GCaMP6f EMP vs. CA1 GCaMP6f median. For median values vs. individual first frames, Wilcoxon rank sum test, p = 0.062 for LIV median vs. LIV 1st frame; p = 0.072 for CA1 jRCaMP1a median vs. CA1 jRCaMP1a 1st frame; p = 0.082 for CA1 GCaMP6f median vs. CA1 GCaMP6f 1st frame. N = 121 for LIV, N = 33 for CA1 jRCaMP1a and for CA1 GCaMP6f. The central line in all boxplots in (a) and (b) is the median, the bounds of the boxes are the 75th and 25th percentiles (i.e., the interquartile range (IQR)), and the whiskers correspond to the highest value or lowest value of the distribution. If the lowest or highest values are outliers (i.e., > 1.5 * IQR from the bounds of the boxes) the whiskers correspond to 1.5 * IQR. Outliers are represented as diamonds. **c-f)** Individual frame (c), median projection (d), global sharpened image (e), and EMP (f) for a representative LIV t-series. **g)** Distribution of absolute fluorescence intensity values for individual first frames (grey), median projection (green), and EMP images (red) for a representative LIV t-series. **h)** GT annotation (magenta) on a representative EMP.

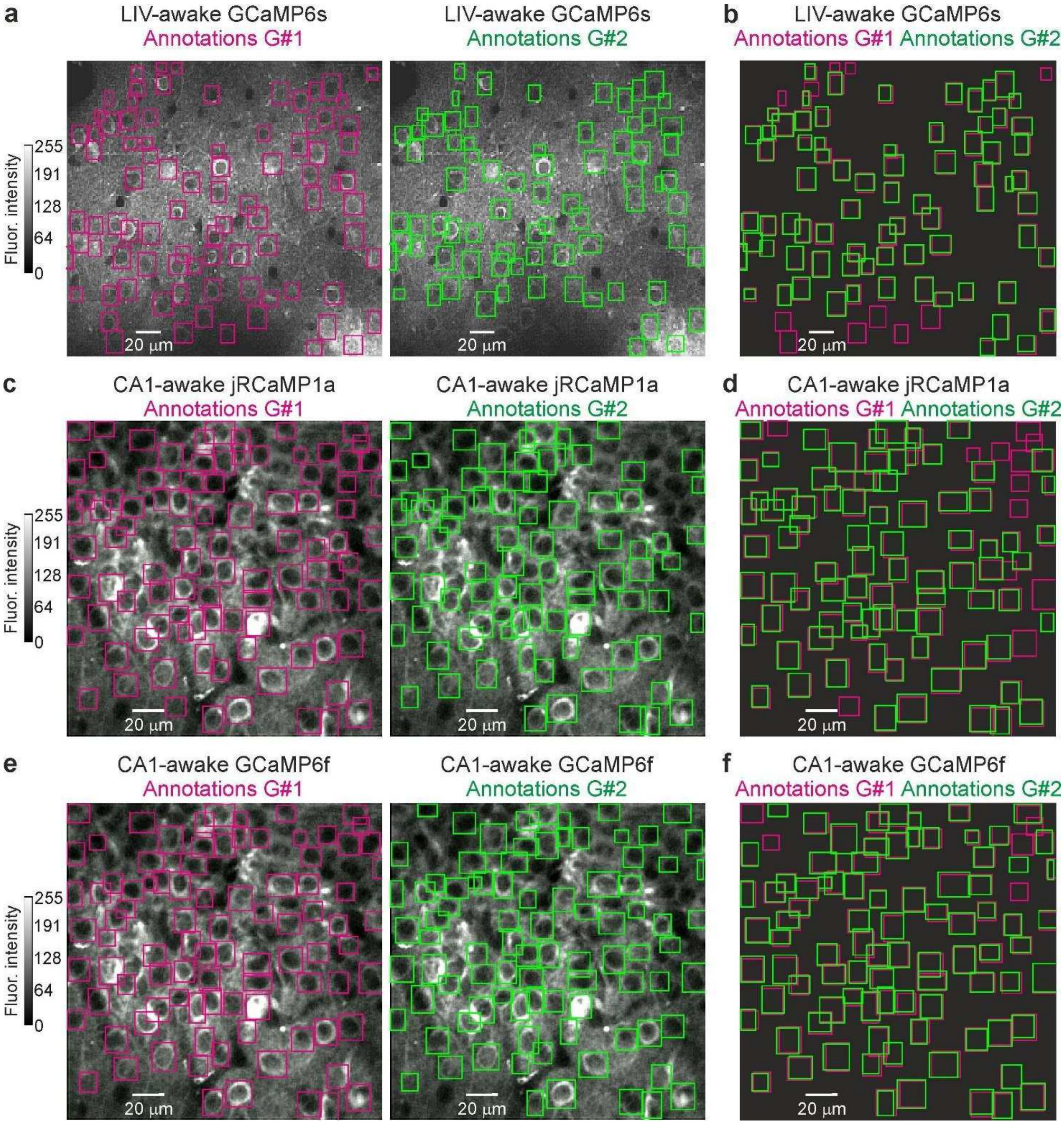

Supplementary Figure 2. Graders' annotations on LIV and CA1 validation t-series. a) EMP image from a representative LIV t-series. Bounding boxes generated by grader #1 are shown in magenta in the left panel. Those generated by grader #2 are shown in green in the right panel. **b)** Superposition of the bounding boxes generated by grader #1 (magenta) and grader #2 (green). **c-d)** Same as in (a-b) for a representative CA1 jRCaMP1a t-series. **e-f)** Same as in (a-b) for a representative CA1 GCaMP6f t-series.

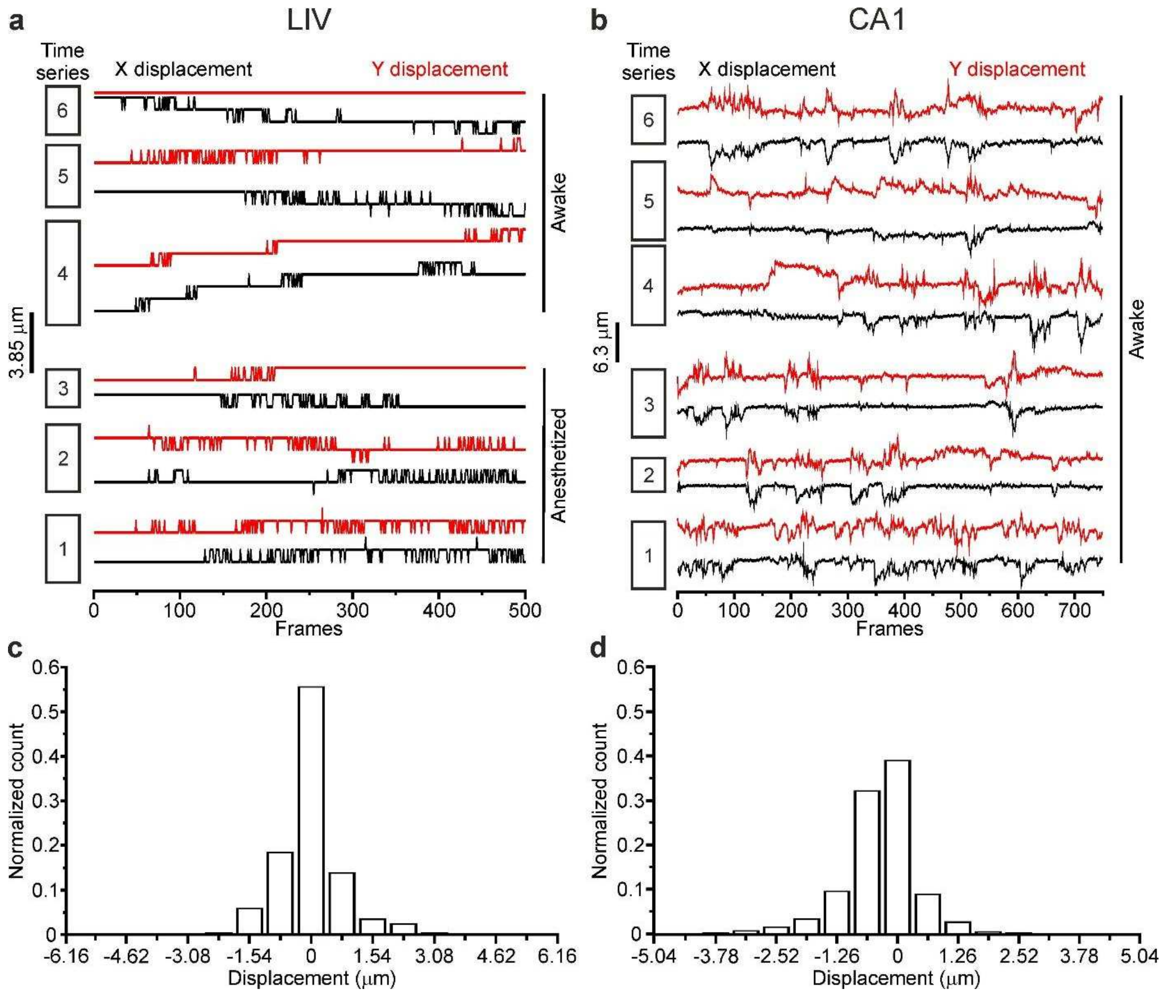

Supplementary Figure 3. **Motion artifacts in the LIV and CA1 validation datasets**. **a)** X, Y displacement of the FOV (black, X; red, Y) expressed in microns as observed across frames of six representative 500 frame-long LIV t-series (traces 1-3 from anesthetized animals, traces 4-6 from awake mice). **b)** Same as in (a) but for six representative 750 frame-long CA1 jRCaMP1a acquisitions. **c)** Distribution of total X, Y displacements in LIV validation t-series ($N = 13$). **d)** Same as in (c) for CA1 jRCaMP1a validation t-series ($N = 12$).

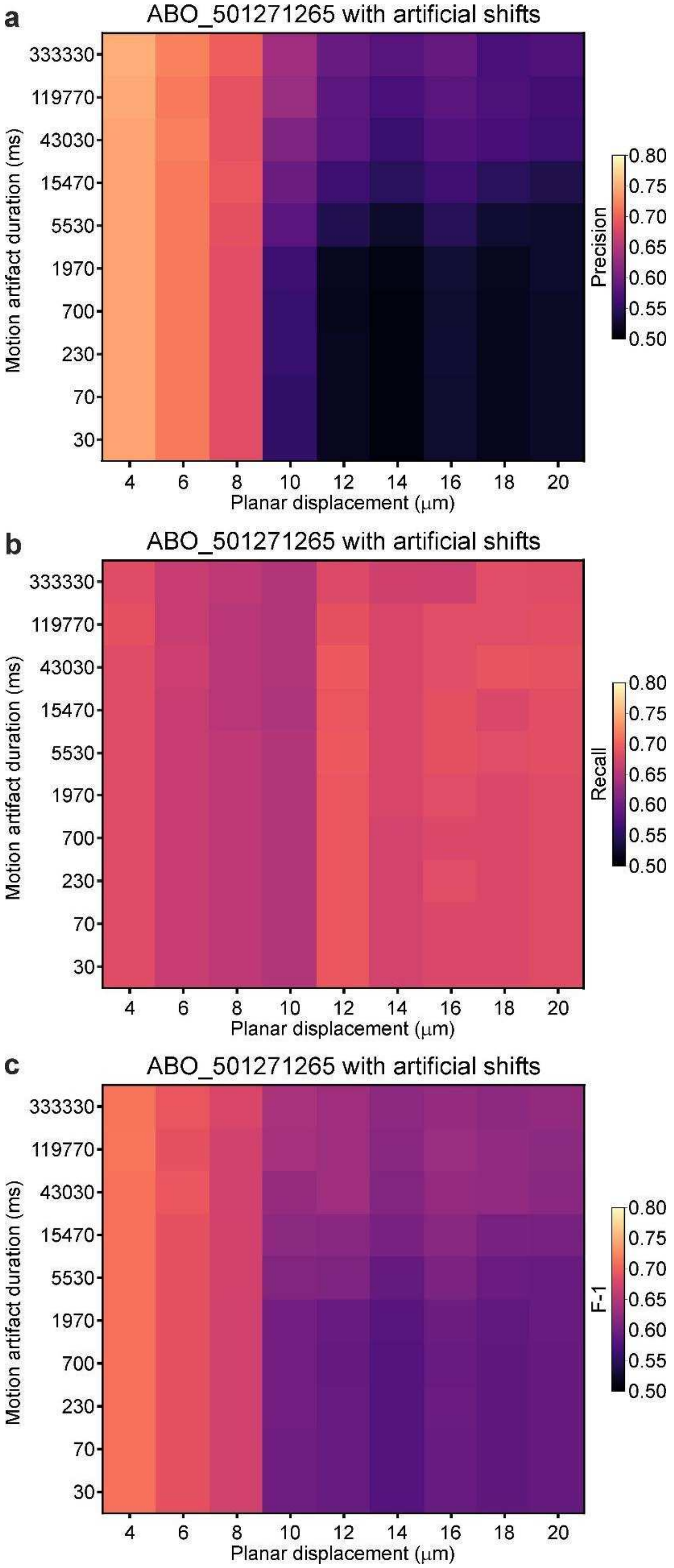

Supplementary Figure 4. Effect of artificial motion artifacts on CITE-On cell detection performance. a) Cell detection precision of CITE-On online for a representative Allen Brain Observatory (ABO) time series (ABO#501271265) as a function of the magnitude and the duration

of the artificial planar displacement. **b-c)** Same as in (a), but for Recall (b) and F-1 score (c). Metrics reported were obtained at the end of the acquisition.

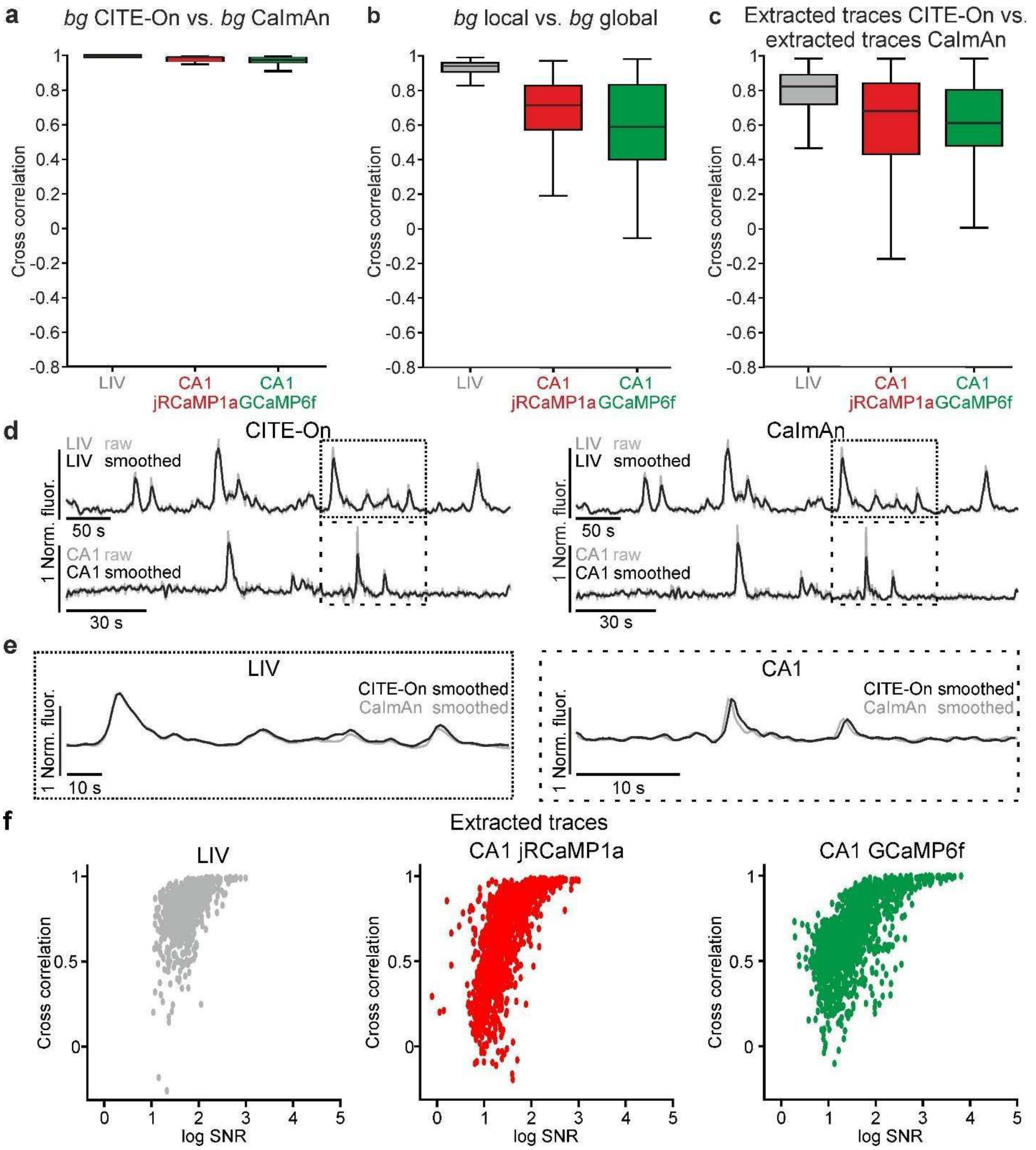

Supplementary Figure 5. Trace extraction from the validation dataset: CITE-On vs. seeded-CaImAn. a) Boxplots showing cross correlation values between background (*bg*) traces obtained using CITE-On and seeded-CaImAn for the LIV, CA1 jRCaMP1a, and CA1 GCaMP6f datasets (N = 13, N = 12, and N = 12 t-series, respectively). **b)** Boxplots showing cross correlation values between global and local background traces computed with CITE-On for the LIV, CA1 jRCaMP1a, and CA1 GCaMP6f datasets (N = 13, N = 12, and N = 12 t-series, respectively). **c)** Boxplots showing cross correlation of background-subtracted and smoothed functional traces extracted with CITE-On and with seeded-CaImAn for all true positive detected identities in the LIV, CA1 jRCaMP1a, and CA1 GCaMP6f datasets (N = 13, N = 12, and N = 12 t-series, respectively). The central line in all boxplots in (a) and (b) is the median, the bounds of the boxes are the 75th and 25th percentiles (i.e., the interquartile range (IQR)), and the whiskers correspond to the highest value or lowest value of the distribution. If the lowest or highest values are outliers (i.e., > 1.5 * IQR from the bounds of the

boxes) the whiskers correspond to 1.5 * IQR. No outliers were present in these distributions. **d)** Representative background subtracted functional traces (grey, raw traces; black, smoothed traces) extracted with CITE-On (left) and seeded-CaImAn (right) from LIV (top) and CA1 jRCaMP1a (bottom) t-series. **e)** Zoom in of the trace portion highlighted in d for smoothed traces extracted with CITE-On (black) and CaImAn (grey) for LIV (left) and CA1 (right). **f)** Cross correlation between background-subtracted and smoothed functional traces extracted with CITE-On and with seeded-CaImAn as a function of the cell's SNR for all true positive identities in the LIV (left), CA1 jRCaMP1a (middle), and CA1 GCaMP6f (right) datasets. Each dot represents a cell detected by CITE-On (see Supplementary Table 1).

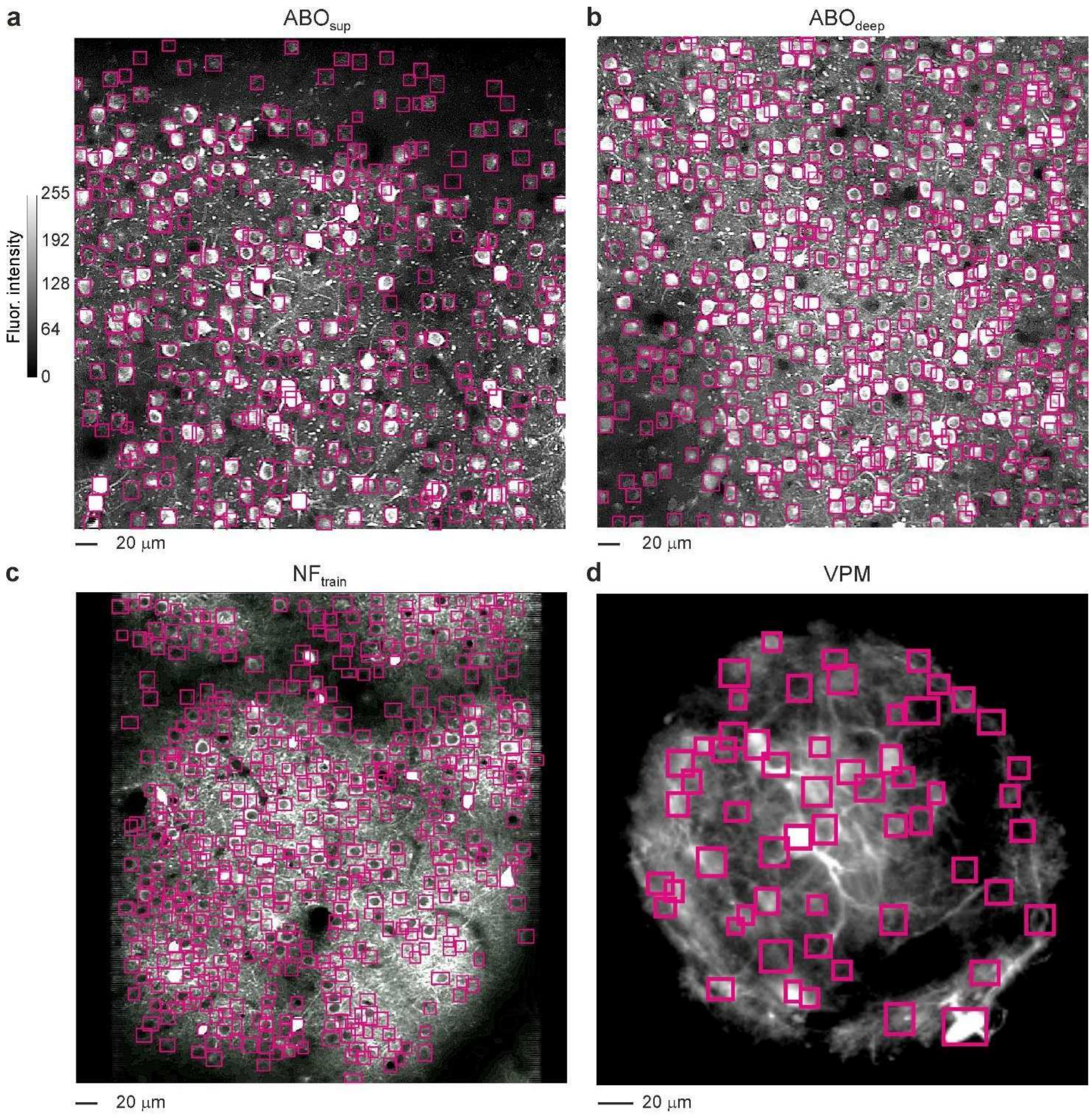

Supplementary Figure 6. Consensus GT bounding box annotation of publicly available datasets. a-d) Median projection of t-series from ABO$_{sup}$ (a), ABO$_{deep}$ (b), Neurofinder (NF) NF$_{train}$ (c), and VPM (d) datasets. Bounding boxes of the consensus GT are shown in magenta.

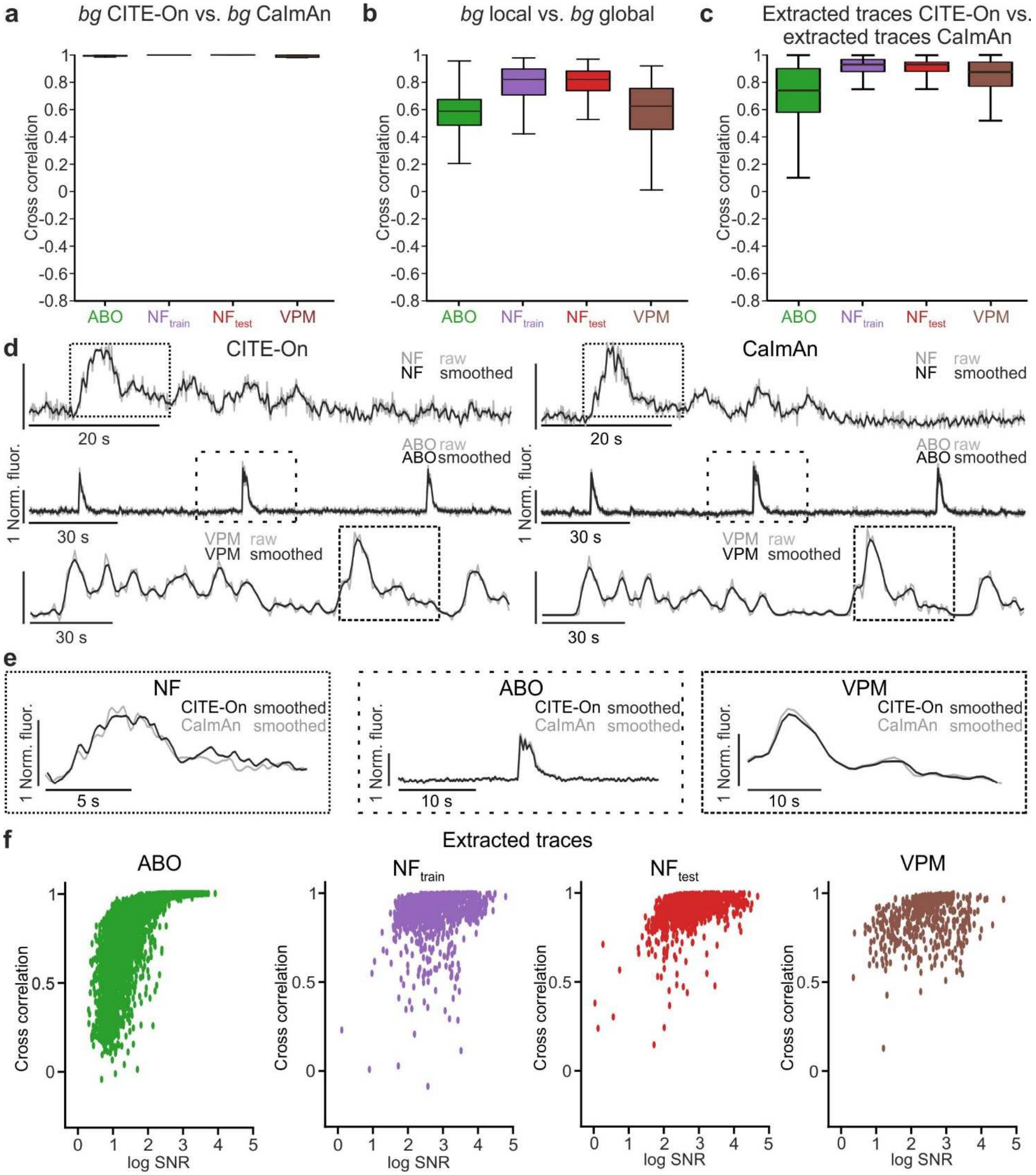

Supplementary Figure 7. Trace extraction from publicly available datasets: CITE-On vs. seeded-CaImAn. a) Boxplots showing cross correlation values between background (*bg*) traces obtained using CITE-On and seeded-CaImAn for the for ABO, NF_{train}, NF_{test}, and VPM data ($N = 19$, $N = 19$, $N = 9$, and $N = 9$ t-series, respectively). **b)** Boxplots showing cross correlation values between global and local background signals computed with CITE-On for ABO, NF_{train}, NF_{test}, and VPM data ($N = 19$, $N = 19$, $N = 9$, and $N = 9$ t-series, respectively). **c)** Boxplot showing cross correlation of background-subtracted and smoothed functional traces extracted with CITE-On and with seeded-CaImAn for the for ABO, NF_{train}, NF_{test}, and VPM data ($N = 19$, $N = 19$, $N = 9$, and $N = 9$ t-series, respectively). The central line in all boxplots in (a) and (b) is the median, the bounds of the boxes are the 75th and 25th percentiles (i.e., the interquartile range (IQR)), and the whiskers correspond to the

highest value or lowest value of the distribution. If the lowest or highest values are outliers (i.e., > 1.5 * IQR from the bounds of the boxes) the whiskers correspond to 1.5 * IQR. No outliers were present in these distributions. **d)** Representative background subtracted functional traces (grey, raw traces; black, smoothed traces) extracted with CITE-On (left) and seeded-CaImAn (right) for NF (top), ABO (middle) and VPM (bottom) acquisitions. **e)** Zoom in of the trace portion highlighted in d for smoothed traces extracted with CITE-On (black) and CaImAn (grey) for NF (left), ABO (middle), and VPM (right). **f)** Cross correlation of background-subtracted and smoothed functional traces extracted with seeded-CaImAn and CITE-On as a function of the cell's SNR for all true positive identities in the ABO (leftmost), NF_{train} (middle left), NF_{test} (middle right) and VPM (rightmost) acquisitions. Each dot represents a cell detected by CITE-On (see Supplementary Table 1).

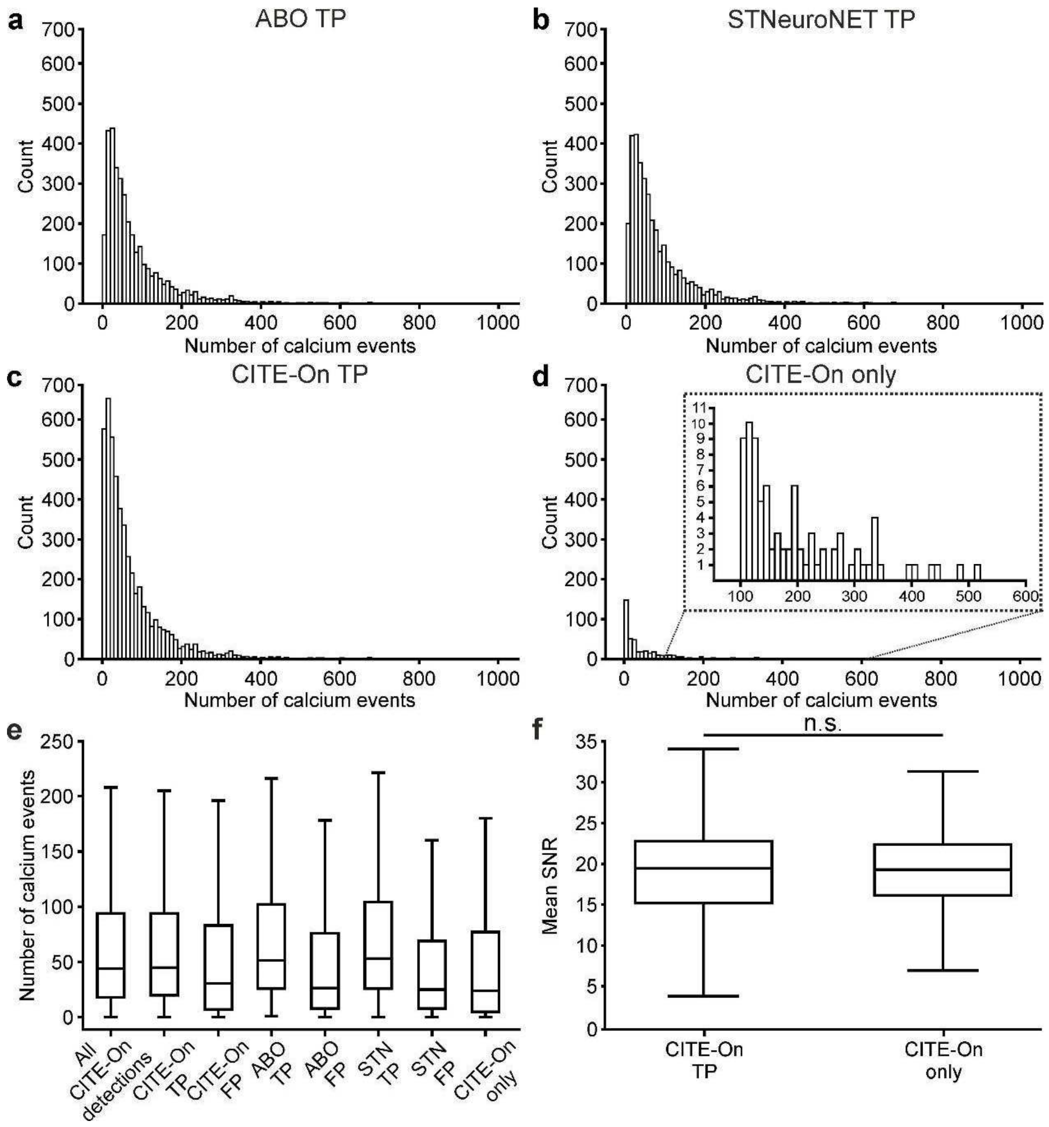

Supplementary Figure 8. Calcium activity in CITE-On, ABO, and STNeuroNET detections. a-d) Distribution of the number of calcium events in true positive (TP) detections in a representative ABO t-series analyzed by ABO (a), STNeuroNET (b), CITE-On (c). (d) Shows the distribution of CITE-On only true positive detections. Data refers to the corresponding GT annotation provided by the consensus GT generated in this work (CITE-On ground truth), that included in the ABO dataset (ABO ground truth)), and that described in Soltanian-Zadeh et al. [22] (STNeuroNET ground truth). **e)** Boxplots showing the number of detected calcium events per cell for all CITE-On detections, CITE-On true positives (CITE-On TP), CITE-On false positives (CITE-On FP), ABO true positives (ABO TP), ABO false positives (ABO FP), STNeuroNET true positives (STN TP), STNeuroNET false positives (STN FP), and CITE-On only true positive (CITE-On only) detections. Data from $N = 19$ t-series of the ABO dataset. **f)** Boxplots of the mean SNR values of all calcium events for all CITE-On true positives (CITE-On TP, left) and for CITE-On only true positive (CITE-On only, right) cells obtained from the same ABO t-series considered in (a-d). One-sided Wilcoxon sum rank test, $p = 0.20$, $N = 4934$ for all CITE-On TP cells and $N = 439$ for CITE-On only cells. The central line in all boxplots in (a) and (b) is the median, the bounds of the boxes are the 75th and 25th percentiles (i.e., the interquartile range (IQR)), and the whiskers correspond to the highest value or lowest value of the distribution. If the lowest or highest values are outliers (i.e., > 1.5 * IQR from the bounds of the boxes) the whiskers correspond to 1.5 * IQR. No outliers were present in these distributions.

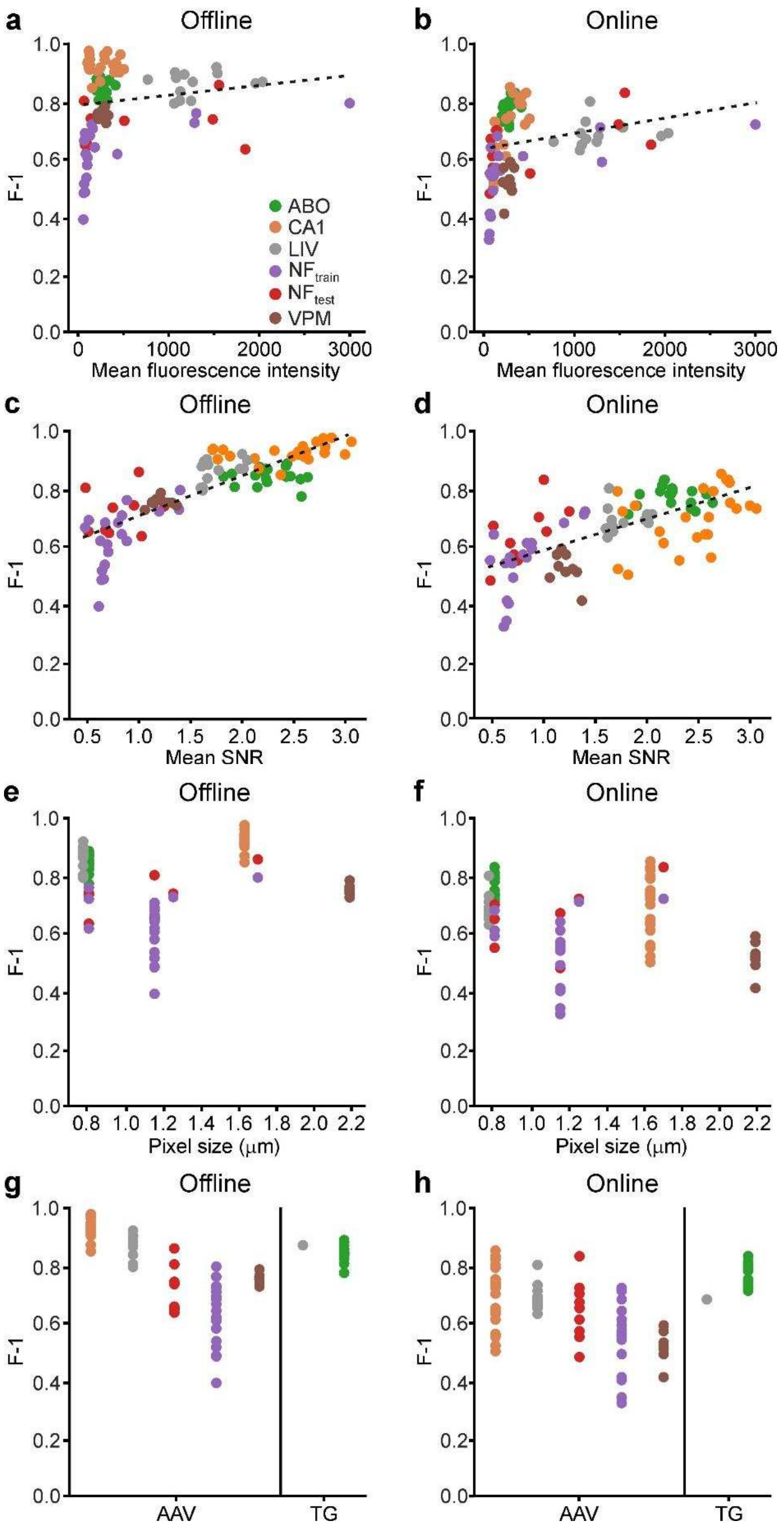

Supplementary Figure 9. Effect of experimental parameters on CITE-On cell detection performance. a-h) F-1 score as a function of mean fluorescence intensity (a-b), mean SNR (c-d), pixel size (e-f), and calcium indicator expression strategy (g-h) for each t-series. Every dot is a t-series and it is color-coded according to the dataset it belongs to. The color code in a applies to b-h. F-1 scores are shown for offline performance in panels a, c, e, g and for online performance in panels

b, d, f, h. The black dotted line in panels a-d indicates the linear fit to the data (R^2 was 0.151, 0.243, 0.828, and 0.674, respectively). In (g) AAV = adenoassociated virus; TG = transgenic.

Supplementary tables

Dataset	# of t-series	# of detections grader 1	# of detections grader 2	# of detections overlap	# of detections grader 1 only	# of detections grader 2 only	# of detections consensus ground truth
ABO	19	6225	5456	5443	782	13	6238
NF_{train}	19	11171	9772	9630	1541	142	11313
NF_{test}	9	3187	2814	2793	394	21	3208
VPM	9	517	470	444	73	26	543
LIV_{test}	13	979	868	861	118	7	986
$CA1_{test}$ jRCaMP1a	12	884	809	795	89	14	898
$CA1_{test}$ GCaMP6f	12	1000	927	916	84	11	1011
LIV_{train}	118	7221	6489	6356	865	133	7354
$CA1_{train}$ jRCaMP1a	21	2123	1856	1833	290	23	2146
$CA1_{train}$ GCaMP6f	21	2218	1963	1940	278	23	2241

Supplementary table 1. Dataset annotation and ground truth generation. Two graders manually annotated the LIV, CA1, ABO, NF, and VPM datasets. The table reports the number of t-series, the number of detections by grader 1 and 2, the number of overlapping detections, the number of detections exclusively produced by grader 1 and grader 2, and the number of detections in the consensus ground truth.

Dataset	mAP	s.d.	*N*	F-1	s.d.	*N*	Precision	s.d.	*N*	Recall	s.d.	*N*
ABO	0.80	0.10	19	0.93	0.02	19	0.998	0.004	19	0.87	0.04	19
NF_{train}	0.71	0.14	19	0.92	0.03	19	0.99	0.02	19	0.86	0.05	19
NF_{test}	0.72	0.10	9	0.93	0.02	9	0.99	0.01	9	0.88	0.04	9
VPM	0.64	0.13	9	0.90	0.03	9	0.95	0.03	9	0.86	0.04	9
LIV_{test}	0.81	0.11	13	0.93	0.03	13	0.99	0.01	13	0.88	0.05	13
$CA1_{test}$ jRCaMP1a	0.76	0.07	12	0.94	0.03	12	0.98	0.02	12	0.90	0.04	12
$CA1_{test}$ GCaMP6f	0.82	0.08	12	0.95	0.01	12	0.99	0.01	12	0.92	0.03	12
LIV_{train}	0.73	0.09	118	0.93	0.02	118	0.98	0.02	118	0.88	0.05	118
$CA1_{train}$ jRCaMP1a	0.74	0.08	21	0.92	0.02	21	0.99	0.01	21	0.87	0.04	21
$CA1_{train}$ GCaMP6f	0.76	0.07	21	0.93	0.02	21	0.99	0.01	21	0.87	0.04	21

Supplementary table 2. Evaluation of consensus GT. The table reports the average (relative s.d. and *N*) of the mean average precision (mAP), the F-1 score, the Precision and Recall across the t-series of the various datasets annotated by the different graders.

Offline												
Dataset	F-1	s.d.	*N*	Precision	s.d.	*N*	Recall	s.d.	*N*	# of TP	# of FP	# of FN
ABO	0.84	0.03	19	0.90	0.06	19	0.80	0.07	19	4934	548	1303
NF_{train}	0.63	0.1	19	0.63	0.09	19	0.62	0.12	19	6849	3932	4120
NF_{test}	0.72	0.07	9	0.74	0.08	9	0.70	0.07	9	2286	716	911
VPM	0.75	0.02	9	0.74	0.06	9	0.77	0.06	9	398	140	123
LIV_{test}	0.86	0.04	13	0.85	0.05	13	0.88	0.05	13	1486	257	205
$CA1_{test}$ jRCaMP1a	0.92	0.04	12	0.88	0.06	12	0.97	0.02	12	1618	222	55
$CA1_{test}$ GCaMP6f	0.93	0.02	12	0.91	0.03	12	0.97	0.02	12	1638	170	56

Supplementary table 3. CITE-On offline cell detection performance. Average (relative s.d. and *N*) F-1 score, Precision, Recall, and number of true positives (TP), false positives (FP), and false negatives (FN) for LIV, CA1, ABO, NF and VPM datasets generated by the CITE-On offline pipeline.

Online												
Dataset	F-1	s.d.	*N*	Precision	s.d.	*N*	Recall	s.d.	*N*	# of TP	# of FP	# of FN
ABO	0.77	0.03	19	0.83	0.04	19	0.73	0.05	19	4573	978	1664
NF_{train}	0.55	0.11	19	0.56	0.11	19	0.54	0.12	19	6164	4906	5026
NF_{test}	0.64	0.10	9	0.68	0.12	9	0.62	0.11	9	2096	921	1101
VPM	0.52	0.05	9	0.60	0.11	9	0.47	0.06	9	244	181	277
LIV_{test}	0.69	0.04	13	0.72	0.05	13	0.66	0.06	13	1127	437	564
$CA1_{test}$ jRCaMP1a	0.73	0.07	12	0.74	0.07	12	0.73	0.10	12	1233	439	440
$CA1_{test}$ GCaMP6f	0.67	0.12	12	0.69	0.12	12	0.67	0.13	12	1145	493	549

Supplementary table 4. CITE-On online cell detection performance. Average (relative s.d. and *N*) F-1 score, Precision, Recall, and number of true positives (TP), false positives (FP), and false negatives (FN) for LIV, CA1, ABO, NF and VPM datasets generated by the CITE-On online pipeline.

ABO Dataset	# of cell detections	s.d.	*N*
All CITE-On	289	21	19
CITE-On TP	260	28	19
CITE-On FP	29	16	19
ABO TP	185	36	19
ABO FP	103	31	19
STNeuroNET TP	190	33	19
STNeuroNET FP	99	24	19

CITE-On only	23	15	19

Supplementary table 5. Cell detections in the ABO dataset. Average (relative s.d. and *N*) number of cell detections in the ABO dataset reported by CITE-On, ABO, and STNeuroNET. TP, true positives; FP, false positives. Data relative to CITE-On were calculated online as the number of identities obtained at the end of the processing of each t-series then averaged across all t-series.
