# Supplementary material for: A deep-learning approach for online cell identification and trace extraction in functional two-photon calcium imaging
Source: Nat Commun. 2022 Mar 22;13:1529. doi: 10.1038/s41467-022-29180-0 (PMC8940911; doi:10.1038/s41467-022-29180-0)
Supplement: Supplementary file 3 — Description of Additional Supplementary Files [file 41467_2022_29180_MOESM3_ESM.docx]

**Description of Additional Supplementary Files:**

**Supplementary Movie 1.** Online tracking of detected identities across the t-series. A representative t-series (# of frames, 750; frame rate, 3 Hz; no motion correction) from the CA1 validation dataset was processed online for cell detection (detection update rate, 10 Hz). Bounding boxes (colored squares) for active detections (i.e. neurons identified by CITE-On in the current frame) and past detections (i.e. neurons identified by CITE-On in previous frames) are represented with or without a central dot, respectively. Each identity was associated with a bounding box color which was retained across the t-series. The position and shape of each bounding box was updated in each frame according to the procedure described in the Results section.

**Supplementary Movie 2.** Frameby-frame manual annotation. Representative t-series (# of frames, 500; frame rate, 1.5 Hz) from the LIV training dataset (awake head-fixed Scnn1a-cre mouse expressing GCaMP6s) used for manual annotation of individual frames. The t-series was motion corrected. Brightness and contrast of each displayed frame of the tseries could be adjusted. Bounding boxes were manually positioned around each visible cell in each frame. Only a minority of neurons were identified in the frame-byframe annotation process.

**Supplementary Movie 3.** Manual annotation of EMP images. EMP image corresponding to the same tseries as in Supplementary movie 2. Brightness and contrast of the EMP image could be regulated to better visualize bright and dim cells. Bounding boxes (green) were manually defined around identified neurons.

**Supplementary Movie 4.** Consensus ground truth on the training dataset. Median projections of the training dataset tseries (CA1 jRCaMP1a, CA1 GCaMP6f, LIV GCaMP6s, LIV Ai95 x Scnn1a-CRE, LIV GCaMP6f and LIV GCaMP7f) are represented in grey. Bounding boxes corresponding to our consensus ground truth annotations are shown in green.

**Supplementary Movie 5.** Consensus ground truth and CITE-On detections on the validation dataset. Median projections of the validation dataset t-series (CA1 jRCaMP1a, CA1 GCaMP6f, LIV GCaMP6s, LIV Ai95 x Scnn1a-CRE, LIV GCaMP6f, LIV GCaMP7f, VPM, ABO, NFtrain and NFtest) are represented in grey. Bounding boxes corresponding to our consensus ground truth are shown in green.

**Supplementary Movie 6.** Online dynamic segmentation and functional trace extraction. a) Raw fluorescence movie from a representative t-series (not motion corrected) in the CA1 jRCaMP1a validation dataset. Detected cells are surrounded by colored bounding boxes and are tracked by CITE-On throughout the t-series. Active detections are identified by bounding boxes containing a dot at their center. Past detections are represented as bounding boxes without the central dot. b) An inset of the same FOV, showing the cell indicated by the white arrow in a. The position of the bounding box (red identity) shifts throughout the movie as a result of the CITE-On tracking process taking place, while planar displacements occur in the acquisition. c) Binary mask produced by the dynamic segmentation process along the acquisition. White pixels represent the segmented pixels, black pixels are discarded. d) Backgroundsubtracted functional trace calculated online throughout the acquisition from the cell displayed in b.
