# Supplementary material for: A deep-learning approach for online cell identification and trace extraction in functional two-photon calcium imaging
Source: Nat Commun. 2022 Mar 22;13:1529. doi: 10.1038/s41467-022-29180-0 (PMC8940911; doi:10.1038/s41467-022-29180-0)
Supplement: Supplementary file 10 — Reporting Summary [file 41467_2022_29180_MOESM10_ESM.pdf]

nature portfolio

Corresponding author(s): Luca Sità, Marco Brondi, Tommaso Fellin

Last updated by author(s): Dec 17, 2021

Reporting Summary

Nature Portfolio wishes to improve the reproducibility of the work that we publish. This form provides structure for consistency and transparency in reporting. For further information on Nature Portfolio policies, see our Editorial Policies and the Editorial Policy Checklist.

Statistics

For all statistical analyses, confirm that the following items are present in the figure legend, table legend, main text, or Methods section.

n/a	Confirmed	
☐	☒	The exact sample size (*n*) for each experimental group/condition, given as a discrete number and unit of measurement
☐	☒	A statement on whether measurements were taken from distinct samples or whether the same sample was measured repeatedly
☐	☒	The statistical test(s) used AND whether they are one- or two-sided<br>*Only common tests should be described solely by name; describe more complex techniques in the Methods section.*
☒	☐	A description of all covariates tested
☐	☒	A description of any assumptions or corrections, such as tests of normality and adjustment for multiple comparisons
☐	☒	A full description of the statistical parameters including central tendency (e.g. means) or other basic estimates (e.g. regression coefficient) AND variation (e.g. standard deviation) or associated estimates of uncertainty (e.g. confidence intervals)
☐	☒	For null hypothesis testing, the test statistic (e.g. *F*, *t*, *r*) with confidence intervals, effect sizes, degrees of freedom and *P* value noted<br>*Give P values as exact values whenever suitable.*
☒	☐	For Bayesian analysis, information on the choice of priors and Markov chain Monte Carlo settings
☒	☐	For hierarchical and complex designs, identification of the appropriate level for tests and full reporting of outcomes
☒	☐	Estimates of effect sizes (e.g. Cohen's *d*, Pearson's *r*), indicating how they were calculated

Our web collection on statistics for biologists contains articles on many of the points above.

Software and code

Policy information about availability of computer code

Data collection	Two-photon imaging was performed using a chameleon ultra II pulsed laser (80 MHz pulse frequency, Coherent Inc, Santa Clara, CA, USA) tuned at 920 nm for GCaMP6/7 imaging and at 990 nm for dual color imaging. Excitation power was 30 - 110 mW as measured under the microscope objective and controlled via a Pockel cell (Conoptics Inc, Danbury CT, USA,). An Ultima II scanhead (Bruker Corporation, Milan, Italy) equipped with 3 mm raster scanning galvanometers (6215H, Cambridge Technology, Bedford, MA) and standard photomultiplier tubes (Hamamatsu Photonics, Tokyo, Japan) and an Ultima Investigator (Bruker Corporation, Milan, Italy), equipped with 6 mm raster scanning galvanometers, movable objective mount, and multi-alkali photomultiplier tubes were used. The three objectives were: 25x/1.05 NA (Olympus Corp., Tokyo, JP) for LIV imaging, 20x/0.5 NA (Zeiss, Oberkochen, Germany) for VPM endoscopic imaging, and 16x/0.8 NA (Nikon, Tokyo, Japan) for CA1 experiments.
Data analysis	All time series preprocessing needed for manual labeling were performed using Fiji (ImageJ V. 1.52p). Manual labeling was performed using LabelImg (version 1.0) (http://github.com/tzutalin/labelImg). The model was trained using the keras implementation of RetinaNet version 0.5.0 (https://github.com/fizyr/keras-retinanet), which contains all the others referenced softwares. All the subsequent analysis was performed with custom Python 3.7 code. All the code presented in this work together with the trained network model and tutorial examples are freely available from our institutional gitlab server at the following link:<br>https://gitlab.iit.it/fellin-public/cite-on

For manuscripts utilizing custom algorithms or software that are central to the research but not yet described in published literature, software must be made available to editors and reviewers. We strongly encourage code deposition in a community repository (e.g. GitHub). See the Nature Portfolio guidelines for submitting code & software for further information.

Data

Policy information about availability of data

All manuscripts must include a data availability statement. This statement should provide the following information, where applicable:

- Accession codes, unique identifiers, or web links for publicly available datasets
- A description of any restrictions on data availability
- For clinical datasets or third party data, please ensure that the statement adheres to our policy

All t-series of our datasets (LIV, CA1, and VPM), our GT annotations for our datasets and for external datasets, and the trained model are freely available at our institutional data repository at the following link:
https://doi.org/10.48557/TRGQOD
Additional publicly available datasets used in this work can be found at the following links:
- Allen Brain Observatory (ABO) visual coding dataset (see Methods section for t-series identification numbers):
https://observatory.brain-map.org/visualcoding
- Neurofinder (NF) challenge dataset:
https://github.com/codeneuro/neurofinder
- Mesoscopic full field imaging t-series:
https://github.com/sofroniewn/2pRAM-paper

Field-specific reporting

Please select the one below that is the best fit for your research. If you are not sure, read the appropriate sections before making your selection.

- [x] Life sciences
- [ ] Behavioural & social sciences
- [ ] Ecological, evolutionary & environmental sciences

For a reference copy of the document with all sections, see nature.com/documents/nr-reporting-summary-flat.pdf

Life sciences study design

All studies must disclose on these points even when the disclosure is negative.

Sample size	The number of samples (N) is reported in the figure legends or in the text. No statistical methods were used to pre-determine sample size, but sample size was chosen based on previous studies (Chen et al., 2013; Miller et al., 2014).
Data exclusions	All recordings with no technical issues were included in the analysis. Animals without sufficient calcium indicator expression were excluded because functional imaging could not be efficiently performed in those mice. This exclusion criterion was pre-established.
Replication	The experiments reported in this work were repeated independently and successfully in three-four animals of both sexes and from different litters.
Randomization	In vivo calcium imaging was performed in different brain regions (cortex, hippocampus, and thalamus) and with different calcium indicators (GCaMP6s, GCaMP6f, GCaMP7f, and jRCaMP1a) in order to assess the validity and robustness of our trained model. Allocation to the different experimental groups was based on these labels (i.e., brain region and type of indicator).
Blinding	Blinding was not used in this study. Blinding was not possible because the same researchers collected and analyzed the in vivo calcium imaging data.

Reporting for specific materials, systems and methods

We require information from authors about some types of materials, experimental systems and methods used in many studies. Here, indicate whether each material, system or method listed is relevant to your study. If you are not sure if a list item applies to your research, read the appropriate section before selecting a response.

Materials & experimental systems

n/a	Involved in the study	
x		Antibodies
x		Eukaryotic cell lines
x		Palaeontology and archaeology
	x	Animals and other organisms
x		Human research participants
x		Clinical data
x		Dual use research of concern

Methods

n/a	Involved in the study	
x		ChIP-seq
x		Flow cytometry
x		MRI-based neuroimaging

Animals and other organisms

Policy information about studies involving animals; ARRIVE guidelines recommended for reporting animal research

Laboratory animals	C57BL/6J mice were purchased from Charles River Laboratories (Calco, Italy; strain code #632), transgenic Scnn1a-cre (B6;C3-Tg (Scnn1a-cre)3Aibs/J; JAX #009613), and Ai95D (B6;129S-Gt(ROSA)26Sortm95.1(CAG-GCaMP6f)Hze/J; JAX #024105) were purchased from Jackson Laboratories (Bar Harbor, USA). Glast-cre-ERT2 (Slc1a3tm1(cre/ERT2)Mgoe; Mori et al., 2006) mice crossed with Ai95D mice were used for CA1 imaging. Adult mice (7-15 weeks) of both sexes were used for experiments.
Wild animals	The study did not involve wild animals.
Field-collected samples	The study did not involve samples collected from the field.
Ethics oversight	All experiments were carried out in accordance with the guidelines of the European Communities Council Directive, and were approved by the National Council on Animal Care of the Italian Ministry of Health (authorization #34/2015-PR, #1134/2015-PR, and #61/2019-PR).

Note that full information on the approval of the study protocol must also be provided in the manuscript.
